# Freedom isn’t free: resource limits on person-centred best interests decisions under the Mental Capacity Act

**DOI:** 10.1093/medlaw/fwag001

**Published:** 2026-03-29

**Authors:** Giles Birchley, Aoife Finnerty

**Affiliations:** Centre for Ethics in Medicine, Bristol Medical School: Population Health Sciences, University of Bristol, Bristol BS8 2PS, United Kingdom; School of Law, Trinity College Dublin, Dublin 2 D02 X376, Ireland

## Abstract

Best interests under the Mental Capacity Act 2005 has been cast as an empowering, person-centred process that protects a person’s rights and freedom of action. In practice this laudable goal is constrained by monetary and temporal resources. Drawing on a qualitative study which encompassed the views of patients, carers, healthcare professionals, and lawyers, we observe that, where resources are inadequate, the quality of decision-making declines and the options on offer are restricted. While austerity has disproportionately disadvantaged people with disabilities and additional needs in numerous ways, in mental capacity law, the impact of this is evident in the gap between the protection of procedural and substantive rights offered by the law. While the courts deal robustly with challenges to ‘faulty’ procedure, challenging substantive issues is difficult and has limited prospects of improving outcomes, even if the decision is clearly inadequate in any sensible interpretation of the court’s aspiration to person-centredness. Tracing these differences back to the different logics of the European Convention on Human Rights and the United Nations Convention on the Rights of Persons with Disabilities, we argue that, as things currently stand, the law cannot resolve these issues, dooming the aspiration to person-centredness to remain constrained and provisional.

## I. INTRODUCTION

‘Freedom is not free’ is a phrase of uncertain origin. While it has become a byline for debates about the balance of counterterrorism and human rights, outside this context, it can frame tensions between any freedom and the cost that freedom incurs.^1^ The Mental Capacity Act 2005 (MCA) was hailed as signifying a ‘move from protection and paternalism to enablement and empowerment’.^2^ Front and centre in guiding decision-makers is the ‘least restrictive’ principle, which seeks to protect ‘the person’s rights and freedom of action’.^3^ Also amongst the core guiding principles is ‘best interests’, an arguably loosely defined concept, which leaves the balance to be struck between freedom and protection to the decision-maker.^4^ Already the common law standard for decision-making,^5^ the centrality of best interests within the MCA can be traced back to the recommendations of the Law Commission in the 1990s. When faced with a choice between a ‘subjective’ test, namely substituted judgment, and an ‘objective’—and arguably ‘*Bolamite’^6^* test—namely, best interests, the Law Commission viewed a best interests test with a strong element of substituted judgment to be the best compromise.^7^ In that way, the Law Commission distanced itself^8^ from the earlier common law interpretation, which amounted to best interests being assessed by reference to professional medical opinion (*Bolam*^9^).^10^

This subjectification of best interests was advanced through a series of autonomy-promoting measures in section 4 of the MCA, including the foregrounding of the participation of the person (‘P’) in decision-making,^11^ consideration of P’s wishes, feelings, values, and beliefs,^12^ and consultation with those who know and care about P.^13^ As such, the autonomy-promoting parts of section 4, together with the least restrictive principle, mitigate the potential for coercion, paternalism, and generic—or non- individualized—decision-making, which could be risked by a loosely defined test of best interests. We suggest, however, that these subjectifying measures cannot be considered in a vacuum; rather, their efficacy depends in no small part upon the resources available for the care, support, and treatment of those who lack decision-making capacity.

We present data from a qualitative study that explored health and welfare^14^ decisions with individuals, their representatives, health professionals, and lawyers. In doing so, we seek to elucidate the severely constraining influence of resource limitations upon the entire infrastructure of best interests decision-making. Best interests decisions are comprised of procedural and substantive aspects: the procedural aspects are the processes or steps that need to be followed when making a decision. The substantive aspects are the content of the options for the treatment, care, or support of P, relevant to the best interests decision to be made. Our data suggested that both procedural and substantive aspects of best interests are impacted upon by a lack of resources. However, our legal analysis suggests the ability of the person affected to attain a satisfactory^15^ resolution where the deficiency is procedural is markedly different from when the failing is substantive.

A complication of our analysis is that ‘resource’ is both financial and temporal—lack of money can manifest in too few staff having too much to do in the time available. Because lack of time is a qualitative judgment, the accusation that professionals falsely profess a lack of time to cover their own failings is frequently repeated in hostile governmental and press briefings.^16^ Nevertheless, within our dataset, patients and members of all professional groups all observed negative impacts of shortages of time upon practice. Inadequate health and social care staffing levels are also corroborated by numerous sources, including high vacancy rates,^17^ longitudinal staff surveys,^18^ and public sentiment.^19^ For this reason, our article accepts that time shortages are a plausible cause of care failings, notwithstanding that inefficiencies or ethical failures might also be present.

This article begins by briefly setting out the context within which the empirical findings are situated. Next, there is a discussion of the relevant methodology, followed by the presentation of data which reveals the impact of resource shortage on best interests decision-making from the perspective of those with personal and professional experience. The contribution then moves to the key areas of discussion: we explore the legal framework, in particular how the courts approach substantive and procedural issues within best interests decisions, highlighting the difficulties this difference poses for people who lack capacity seeking to challenge failures in substantive aspects through the courts, including on human rights grounds. We then argue that the extent to which the MCA’s aim of ‘enablement and empowerment’^20^ can move beyond a rhetorical claim^21^ will depend, at least in part, on what resources are provided. We suggest that, while person-centredness is central to the current conception of the MCA, the difficulty in challenging substantive issues means that the person is not central in the way they should be.

The roots of our claim lie in the widespread recognition of the disproportionate effects of austerity policy on those with disabilities. Addressing such disproportionality through relevant legal mechanisms has proved challenging, despite concerns that the disproportionate effects of austerity betray an ideological basis.^22^ We conclude that this situation suggests that austerity has eroded the MCA’s potential for empowerment and enablement inasmuch as the law, at least formally, effectively allows person-centredness to exist in the realm of procedure and process but not in the lived reality of substantive wellbeing. We explore some of the reasons why this might be the case before ultimately concluding that until this situation changes, there will be a dissonance between many best interests decisions and the aspirations that the law espouses for genuine person-centredness in best interests decision-making.

Finally, while lack of resourcing in health and social care, and consequently best interests, may be considered ‘common knowledge’ in many circles, we suggest that it is vital to substantiate both the deleterious effect of resource shortages on vulnerable adults and their loved ones, and the role of the law in addressing these impacts. In doing so, we do not claim that a lack of resources is the only impediment to genuine person-centredness, nor do we argue that persons with capacity are somehow undeserving of resources. We accept that poor decision-making can arise in well-resourced environments, and that deficiencies in substantive aspects of best interests decision-making are only part of wider patterns of injustice. Indeed, those who lack capacity could arguably be said to have a more advanced legal infrastructure and ability to challenge issues than other marginalized groups (eg those with marginal capacity and the unfriended).^23^ Addressing this urgent issue in its widest sense is not something we attempt here, although we believe our analysis can usefully contribute.

## II. CONTEXT

### A. Best interests

‘Best interests’ is an important concept within health and social care decision-making. However, because it is the standard that applies to a wide range of different situations and circumstances,^24^ it is deliberately under-specified. This under-specification means that ‘best interests’ has been described as ‘empty’,^25^ requiring other perspectives to take shape for each individual. These can include professional, philosophical, political, and human rights perspectives.^26^ As outlined in the introduction, best interests were historically assessed by reference to professional (medical) opinion.^27^ Being so intertwined with a Bolamite^28^ notion of the importance of professional opinion, best interests held few, if any, protections for P and gave scant regard for P’s own opinions on the matter being deliberated.^29^

The MCA marked a significant change in this approach, with best interests under the MCA encompassing a range of factors including P’s past and present wishes and feelings, as well as their and beliefs and values.^30^ As indicated in the introduction, the MCA also contains the ‘least restrictive’ principle,^31^ which serves as a reminder that the freedom of action of people who lack capacity is ‘as important to them as it is to anyone else’.^32^ One role of the specialist court, which oversees the MCA—the Court of Protection (COP) —consistent with section 1(6) has been articulated in *Wye Valley NHS Trust v B^33^* as that of preserving and promoting the ‘freedom of action’ of persons with disabilities ‘to control their own lives’.^34^ The importance of P’s wishes, feelings, values, and beliefs to a best interests assessment, together with the prominence of the ‘least restrictive’ principle, putatively endorses an approach which upholds, or at least minimizes, interference with the freedoms of individuals. However, how effective the COP can be in the role articulated in *Wye Valley* with respect to people with disabilities is debatable.

On one hand, the very legal framework underpinning much of the activity of the Court—‘best interests’ under the MCA—has been considered non-compliant with Article 12 of the United Nations Convention on the Rights of Persons with Disabilities (UNCRPD),^35^ which guarantees equal recognition before the law. The UNCRPD Committee, which is the body tasked with monitoring the implementation of the Convention by states, has expressed the non-binding view that ‘best interests’ is incompatible with Article 12, arguing instead that it should be scrapped in favour of a ‘rights, will and preferences’^36^ standard.^37^ There are mixed views regarding this position^38^; however, specifically in relation to best interests in the MCA, the Essex Autonomy Project (EAP) rejected the need for a change of standard in order to achieve UNCRPD compliance.^39^ Rather, the EAP recommended reform of the MCA to give precedence to wishes and feelings within the best interests framework in the form of a rebuttable presumption that best interests decisions should accord with the wishes of the person if they can be reasonably ascertained.

On the other hand, the commitment to upholding the rights and freedoms of people with disabilities shown in cases such as *Wye Valley* shows some consistency with the model of disability and ethos contained in the UNCRPD. This ethos has been described as adopting a vision ‘where non-discrimination … is a core value’ and as ‘the re-articulation of rights found in other treaties in ways that [would] make those rights meaningful to people with disabilities’.^40^ While the UNCRPD is not part of domestic law, and is not directly applicable,^41^ Lawson and Series suggest that judgments may carry its implicit influence, even if they fail to explicitly cite it, given its persuasive influence on the common law.^42^ More explicit influence has been exerted by the European Convention on Human Rights (ECHR), directly applicable to English law since the adoption of the Human Rights Act 1998. The ECHR has often been called upon to uphold P’s freedoms,^43^ although the ‘different normative anchors’^44^ of the ECHR and the CRPD have been noted.

How consistently the Court of Protection can and does promote the freedom of action of people who lack capacity through the legal process remains questionable.^45^ We suggest that a clear distinction can be drawn between the remedies available to those who encounter improper or deficient best interests procedures^46^ and those who suffer infringements on substantive freedoms because resources are not forthcoming to provide better options.^47^ These different approaches cannot be explained without understanding the tensions arising between economic policy and a putative person-centred role of the law in this area. These tensions will be explored in more detail in Section V.

### B. Austerity

Best interests and resource constraints cannot be discussed in a vacuum. Mediocre or poor options will be commonplace so long as these decisions take place amid resource scarcity within social care and health that is hitherto unprecedented.^48^ Over the decade 2010–20, austerity in the UK included sharp declines in resources for health and welfare that disproportionately affected people with disabilities. Despite demand increasing, the number of people receiving home care visits fell by 30 per cent between 2010 and 2014,^49^ and fewer families who provide care received financial or in-kind support.^50^ Total social care spending did not return to 2010 levels until 2020.^51^ Local Authorities, who provide the majority of social care, lost around 40 per cent of their funding between 2010 and 2020.^52^ As authorities desperately try to stave off bankruptcy,^53^ they may be incentivized, or perhaps forced, to radically restrict the options offered to those who still qualify for help.^54^ Since health is largely socially determined, squeezes on social care funding also increase demand on healthcare.^55^ Essentially flat healthcare spending failed to keep pace with this demand, meaning that the social care crisis played a large part in leaving the NHS, even before the COVID-19 pandemic, ‘under resourced, understaffed and woefully underprepared’ for the challenges it faces.^56^ Despite widespread evidence of the negative impact of austerity policy on people with disabilities and those with additional care needs,^57^ conditions seem unlikely to improve,^58^ despite austerity officially ending in 2019.^59^

## III. METHODOLOGY

The ‘Balancing Best Interests in Healthcare Ethics and Law’ (BABEL) study examines best interests decisions in healthcare, ethics, and law. Two workstreams gathered empirical data^60^ from stakeholders in healthcare (WS1) and law (WS2). The study thereby gained insights into more ‘everyday’ best interests decisions, in addition to those formal decisions taken by the COP, reasoning this would give a more complete picture of best interests.

After approval by the University of Bristol’s Health Sciences Research Ethics Committee (WS1) and Law School Research Ethics Committee (WS2), we recruited patients, relatives, and healthcare professionals (WS1) and lawyers (WS2). WS1 participants were recruited for their experience of best interest decisions in healthcare settings, although these decisions frequently elided into social care.^61^ WS2 participants regularly appeared in the COP and/or the High Court, with almost all having experience in both healthcare and welfare matters.^62^ All participants gave their informed consent to be interviewed, and for anonymized quotes from the interviews to be published.

Sixty-eight respondents participated in WS1 interviews, focus groups, and ethics discussion groups. To focus on those whose decisions were governed by the MCA, we have excluded data from children and parents, reporting here on 27 participants (17 interviews, 7 in focus groups, and 3 in ethics discussion groups). Participants consisted of health professionals, patients, and relatives, many of whom were welfare attorneys (LPA—Lasting Power of Attorney under MCA, sections 9–14). Fifty-two participants took part in WS2, and 38 of those were included in this analysis. We do not specify the occupation of the lawyer to protect the anonymity of participants, using the generic term ‘lawyer’ to denote both barristers and solicitors. Participants are identified by a code to indicate WS1 interviewee (**I**XXX), focus group participant (**FG**XXX) or ethics discussion group participant (**EDG**XX) or WS2 participant (**2**XX).

Data was collected between October 2021 and October 2023. All interviews and focus groups were semi-structured and qualitative in nature and followed a topic guide, which was periodically reviewed and revised as needed. WS1 performed successive data collection activities. We initially conducted interviews, drawing anonymized vignettes from interview participants’ stories. Focus groups with new participants were centred around these anonymized vignettes. After further analysis to describe themes in the data, ethics discussion groups were conducted with a third wave of participants to discuss these preliminary themes. At each successive stage, the questions and discussion sought to approach issues in a more abstract way, so that while interviews discussed individual cases, focus groups and ethics discussion groups concentrated on general principles. Most interviews and ethics discussion groups, and all focus groups, took place online. Interviews and focus groups were audio-recorded, transcribed, and analysed using the reflexive thematic method.^63^ WS1 and WS2 analyses took place separately. Analysis consisted of inferences about identified elements (‘codes’) within interview data of diverse participants, allowing development and comparison of themes. Presentation of findings between WS1 and WS2 alerted us to the significance of resources in the decision-making process in both datasets, which also allowed the authors to draw from core themes identified in both workstreams—‘barriers’ from WS1 and the ‘constraint’ theme from WS2—for this contribution. While resources were significant in decision-making pertaining to both adults and children, we discuss only adult^64^ best interests here.

## IV. EMPIRICAL RESULTS

As outlined in the introduction, empirical data from both WS1 and WS2 suggest that lack of resources affects both substantive and procedural aspects of best interests decision-making. Substantive aspects within the welfare context are often manifested in the options, or lack thereof, for those with additional support or care needs. These options were either limited by the relevant public bodies, or only a single option was offered. Substantive aspects of best interests in healthcare contexts, though less apparent within our data, did appear to be an issue because available staff and resources could not meet demand for the basics of care at times.

In terms of the procedural aspects of best interests, within healthcare, procedures suffered because clinical areas were understaffed, leaving little professional time for detailed engagement with the person or their family. Constrained specialist services were less accessible to some patients, leading to greater demand on emergency admissions, which were intrinsically unsuited to individualized best interests assessments. In a welfare context, the suggestion was similar: understaffing and excessive caseloads led to individuals receiving a superficial best interests process, and it was only through the person, family, or advocates objecting or the commencement of legal proceedings that a more thorough and individualized process occurred.

Unsurprisingly, both procedural and substantive deficiencies in best interests were identified as being causes of legal disputes. Furthermore, resource shortages were so endemic that they were generally accepted as norms, though family members often refused to accept that options were limited for their loved one, perhaps indicating a dissonance between the general and the specific. Resource shortage is understood as a general issue that affects health and social care, but is rejected as a reason for poor options or processes when it impacts upon a specific person. We will now consider substantive and procedural aspects in more detail with reference to our data.

### A. Substantive

Resource constraints were observed as significantly impacting upon the quality or availability of options in the course of best interests decision-making. Participants from both WS1 (health professionals and patients) and WS2 (lawyers) offered both general observations about the finitude of funding, and specific discussion of the impact of resource constraints on the substantive aspect of best interests. As lawyer 206 said:

*I have got cases where people have been detained in hospital for decades. They lack capacity to make the decision about accommodation and care, but there isn’t, literally, a care provider in the community that is able and willing to put forward arrangements for them. So, it is either because of a lack of … suitable care provider, or, more commonly, it is a lack of money. Because, obviously, better options cost more.*

From a clinical perspective, resource scarcity was so endemic within all clinical activities that it was described akin to a law of nature. In one striking example, I028, a nurse consultant, analogized financial budgets to ‘environmental limits’; inherent limitations on what could be done that just needed to be accepted:

*I think we all understand that we’re trying to act in a way that is good for that individual, but it’s always bound by what resources you’ve got … What might be in somebody’s best interests might wipe out the entire NHS budget, mightn’t it? So there are environmental limits on that, aren’t there, and resource limits?*

There is a chasm between the extreme identified here—options that might ‘wipe out’ budgets—and the issue in respect of many best interests decisions, namely the need for *better* or less restrictive options for care. It is the latter with which this article is concerned and upon which many participants commented. Moreover, while the resource limitations found in the delivery of care and in society at large resemble the finite limits set by natural resources, they are not identical to these natural limits because they result from policy choices. As constraints on freedoms, they are thus what Adorno described as a ‘second nature’,^65^ policy choices that present barriers to the individual that are so immutable that they may be confused with natural, rather than ideological, limits. On the other hand, Lawyer 216 recognized the source of constraint as a policy choice, suggesting that the effectiveness of the MCA fell down because:

*[W]e as a society apparently don’t seem prepared to either put the sufficient resources in to do it well or to run the system in such a way that the existing resources are used profitably because of [the government].*

We will come back to this point about ideological policy choices in the discussion.

Lack of resources for suitable options was identified by lawyers as a driver of legal disputes, with observations that the ‘patchiness’ or ‘inadequacy’ of state-provided care for adults with additional needs ‘leads to a lot of disputes’ (225). Shortages were also a source of frustration for lawyers themselves, with one claiming that equating elderly people unwillingly residing in care homes because of lack of home care funding with ‘what was best’ was ‘insulting’, instead suggesting best interests should be termed ‘least worst option’ (217). Such interpretations of best interests arose because resource constraints had so severely limited the options available to decision-makers. Arguably, the reality of people’s professional and personal experiences sets a very different tone to the putatively empowering ethos of the MCA. In practice, empowerment and freedom seem unaffordable and unrealistic.

Resource shortages, or their impact, also appeared to be a topic to be approached with the utmost care in discussions with family members. While families may be aware of, and perhaps even sympathetic to, the idea that limits are imposed by resource scarcity rather than health professionals, when these limits were overtly raised in best interests discussions, they could be met with affront. An LPA recounted how she had berated a clinician who had signalled that the hospital’s difficulties in finding appropriate in-patient care for her mother indicated discharge was the most appropriate response:

*I was not having any nonsense from this guy. I said, "She’s not safe at home. She needs more support, and you need to admit her into a bed, and that is the safest place for my mother, until somebody can have this meeting." So, he was a bit shirty with me, and he said, "Oh, we’ll see if we can find a care of the elderly bed." And basically, told me, the hospital, I was putting their hospital under stress because I wouldn’t come and pick her up … Their interest was just chucking her back out again. (I047)*

Resources, then, seemed both influential in professionals’ approaches to best interests decision-making and potentially incendiary if broached in discussions of best interests. Thus, resource limitations appear to have a corrosive impact not only on the ability to offer suitable options but also on dealings between professionals and families. Furthermore, although our focus is on the impact of lack of resources on the ‘freedom’ and ‘empowerment’ aspects of best interests, here we see that shortages can also affect the ‘protection’ aspect. We suggest that resource constraints push the decision away from balancing freedom and protection in a way that is right for the person, and towards merely making a passable decision.

### B. Procedural

Resource constraints were often identified as factors that reduced the quality and effectiveness of the best interests decision-making process. Participants from all groups concurred that lack of professional time was a significant barrier to optimal best interests decision-making processes, or in some cases, to these processes being followed at all. Given that the best interests process was acknowledged by lawyers as one which ‘really requires quite a lot of detailed analysis and consideration’ and time to be ‘done properly’ (224), most often these constraints manifested in limited professional time to engage with patients and those close to them to the extent necessary. Finding the time needed was identified as a constant challenge within fast-paced clinical environments. Within the welfare context, the significant pressures that social workers were under were identified by Lawyer 225 as impacting on the best interests process:

*[Y]ou look and you think, “Well, a social worker’s got a big caseload of people, so maybe they haven’t thought about all this as they should have.” And I’ve seen tons and tons of cases where … you expect the local authority social worker with a big legal team … with all the supervision and support that they’ve got, will kind of be getting everything right, will have remembered all the facts and will have analysed everything. And it’s very, very rare that they do that … they’re firefighting, they’ve got too many cases, they’ve got colleagues off on the sick all the time, it’s a difficult job at the best of times. So, sometimes that’s the cause of them then being not as knowledgeable about the case as they should be.*

Within healthcare, ‘taking time’ went against a culture that prioritized swift decision-making. While there are many reasons why a decision may need to be made quickly—including, naturally, ensuring the lawful basis for decisions^66^—the need to decide too quickly can create a tension between time intensive individualized best interests processes and professional and cultural norms. A junior doctor, I044, suggested that pressure to ensure the most efficient deployment of treatment resources often motivated decisions:

*I think the modern NHS, often time is a big issue … there’s sometimes, I feel, there’s quite a lot of pressure to make the decisions very quickly, because if there are 20 patients on a ward, and you have to make a decision quite rapidly, are we going to treat, are we not going to treat?*

Lack of professional time and short-staffing leaves scope for decision-makers to depart from the best interests process, or for them to lack the necessary skills. I026, an Independent Mental Capacity Advocate (IMCA),^67^ explained that staffing and resource pressures contribute to a lack of appreciation of MCA procedure. Managers may be aware of the MCA obligatory processes, such as consulting widely or involving the patient in the decision,^68^ yet patient-facing professionals may overlook these processes because they are overwhelmed with the demands of basic care, lacking additional time for training:

*Sometimes it’s they’re not thinking it’s a best interest decision. They’re just supporting someone the best they can … the care home managers don’t always seem fully up to speed with the whole process. They, kind of, know it, but the staff aren’t getting it. The staff are too busy being short-staffed, and helping people eat, and doing what they’re doing, so I think some of it is a staffing and a resource issue. … I’m not saying that they’re not acting in, but I don’t think they’re thinking of it like that always, and it’s not filtering down to the day-to-day stuff in the same way. They’re not seeing it in the same way, so it would need a lot more time for training and discussion that they don’t have. (I026)*

In a similar way, time pressures within medical settings were identified, perhaps understandably, as leading to a similar process of (de)prioritization. One lawyer (221) suggested that the focus on getting the medicine ‘right’ left no additional time for the sorts of in-depth conversations that would properly inform a person-centred best interests decision:

*So I think doctors focus on medical best interests … they don’t have enough time to carry out investigation of a more all-encompassing best interests assessment that would allow them to identify really early on specific, highly individual beliefs that might affect or weigh heavily in the balance of best interests….*

The appropriateness of the decision-making setting was affected by wider funding challenges. One paramedic, I031, suggested that the comparatively poor funding of adult services meant that older patients tended to see emergency department clinicians for acute admissions rather than specialists. They observed that access to ‘consultant-level care’ tended to come easier for children, tentatively attributing this to a ‘financial resource element’. A significant implication is that resource limitations indirectly funnelled older patients toward a service that is poorly fitted to the time-intensive and meticulous process of assessing capacity, investigating the person’s wishes and feelings, and consulting widely to inform a best interests decision. As EDG01 (another paramedic) observed, emergency care was poorly suited to the MCA processes. Because of the very nature of emergency intervention, paramedics attending emergencies could not develop the necessary relationships and have calm and considered conversations about P’s wishes. Emergency care was ‘too late [for] the really good communication … and being able to explain everything and have complete understanding between everyone making the decisions’.

Although a certain proportion of best interests decisions will be legally challenged irrespective of how well the decision-maker has adhered to the statutory requirements in the MCA,^69^ poorer quality, inadequate, or non-existent best interests processes in health and social care are certainly a trigger for an application to the COP. Lawyer 228 observed:

*I think probably in about 80% of the Court of Protection cases I see, I feel very strongly that the case could have been resolved much earlier and much more cheaply and with much less damage to relationships if there had been either proper decision making, proper capacity assessment, or mediation—either informal or formal—at a much earlier stage.*

While acknowledging that poor decision-making can happen for a variety of reasons, not limited to resource scarcity, the circular and self-perpetuating nature of resource shortages is worth highlighting. Resource scarcity leads to poorer quality decision-making, which in turn leads to costly court processes, thereby exacerbating the financial pressure upon public bodies. The potential for a further exacerbation of this pressure can be found in the fact that lack of resources and public funding shortages have specifically been rejected as a justification for procedural failings in court proceedings—where a public body fails to comply with orders—with such failures having a significant implication where the decision to award costs against the relevant public body is concerned.^70^

## V. DISCUSSION

The empirical picture presented suggests that resource shortages fuel deficiencies in both the substantive and procedural aspects of best interests decision-making under the MCA. Yet whether scarce resources render these deficiencies *acceptable* from a legal perspective is strongly linked to whether the impact is on the substantive or procedural element. Although the MCA is about practical steps, the interpretation of section 5 of the MCA provides a safeguard to prevent lack of resources being used to justify a shoddy best interests process.^71^ Yet resource constraints can justify the provision of exceptionally poor options.^72^ The courts sometimes profess a commitment to equalizing the rights and freedoms of persons with disabilities^73^; however, the differentiation of process and substantive elements places this commitment in tension with what can and does happen in practice. Dichotomizing the quality of the best interests process and the quality of options available to the person at the centre of that process can therefore at best be viewed as a barrier to what we have termed ‘genuine’ person-centredness in the MCA. Arguably, the roots of this tension can partly be traced to the fallacy that making human rights rhetoric an empowering reality can be done without significant costs. International human rights law has been described as ‘an empty vessel on the domestic political scene’,^74^ requiring political action at a domestic level to be filled; a critical aspect of this domestic action, we suggest, is funding. This gap is egregious in policy,^75^ and can also be said to extend from the limits placed by resource implications upon what the European Court of Human Rights itself can rule.^76^ As such, a wider recognition that freedom is not free is a tangible, if small, first step in addressing this issue.

### A. Resources, best interests, and the Courts 1: substantive aspects

The 2014 House of Lords scrutiny of the MCA contained concerns that in reality ‘resource-led decision-making predominate[s]’.^77^ It is clear, however, that the door to resource-led decision-making has always been wide open. Indeed, one of the perplexing^78^ aspects of this comment is that it fails to acknowledge that the MCA gives no remit to the Court to instruct public bodies as to the options they should provide.^79^ This was explicit in the Law Commission’s report on the draft Mental Capacity Bill, wherein it was stated that ‘the court only has power to make any decision which the person without capacity could have made’, and furthermore that if the person concerned has no power ‘to demand the provision of particular services then the court can do no such thing’ on their behalf.^80^

To the Law Commission, then, the MCA was envisaged as allowing a choice between options offered, rather than offering a venue in which to challenge those options. This position was made clear in *N v ACCG*,^81^ a case which concerned a dispute over the options for aspects of N’s care and contact which were provided by the Clinical Commissioning Group (CCG). The Supreme Court held that the Court of Protection had ‘no greater power to oblige others to do what is best than P would have himself’, concluding that ‘just like P, the court can only choose between the “available options”’.^82^ This is because resource allocation is ultimately a matter of wider economic well-being and a matter for the relevant public body,^83^ not the Court of Protection. As such, any scrutiny of the appropriateness of a public body’s decision was ultimately a matter for judicial review, or if appropriate, the Care Act 2014.^84^ In summarizing the case, the Supreme Court stated that *N v ACCG* ‘was a case in which the court did not have power to order the CCG to fund what the parents wanted [nor the] power to order the actual care providers to do that which they were unwilling or unable to do’.^85^ Ultimately, therefore, as far as best interests determinations are concerned, the funding decisions made by the public body prescribe what the COP can hold to be in the person’s best interests. Whether this was the case in practice as well as in theory prior to the decision in *N v ACCG* is a matter of opinion.^86^

In a critical account, Clough contends that *N v ACCG* shows that ‘the possibility of respecting wishes and feelings through best interests decisions is foreclosed by the institutional decision-making processes earlier on, such as where doctors or social care professionals are not willing to provide certain treatments or services’.^87^ Yet the position in *N* cites,^88^ and is consistent with, the Law Commission’s earlier view,^89^ as well as with an earlier line of precedent on social provision, which holds that ‘needs for services cannot sensibly be assessed without having some regard to the cost of providing them’.^90^ Furthermore, the Law Commission’s view seems consistent with the longstanding position that interference with decisions of public bodies is largely beyond judicial powers.^91^ The limitation of the COP to pronouncements on procedural rights has been argued by Ruck Keene and Cordone to be an example of the private/public law distinction in the MCA.^92^ Whether this distinction is a hard boundary on the activities of the COP is open to question, of course, although outside of the scope of this article.^93^ In any event, the formal power of the COP to address issues of resources is limited in respect of healthcare and welfare matters, in that the COP can require neither medical professionals to provide particular treatment,^94^ nor public bodies to provide more funding or options. This principle has been upheld, even when none of the options have been genuinely satisfactory.^95^ Thus, while increased scrutiny and negotiation created by legal proceedings may make more options apparent, resource limitations prescribe what the Court can hold to be in the person’s best interests.^96^ This is predicated on the expectation that the options offered by the public body have been scrutinized according to their own internal principles, including the restrictions of economic necessity.^97^ The difficulty with this limitation for the people at the centre of such cases is starkly evident at a time when there is widespread consensus that services are in a state of crisis.^98^ In such circumstances, best interests decisions may become a matter of choosing *the least worst option*, despite the perception that the MCA is—or should be—an empowering and enabling instrument that places the person at the centre. Clearly, in order to enable, empower, and do what is best for that person, the resources need to be available to fund both the procedural and substantive aspects of that endeavour.

While appeal mechanisms are available, the ability of the person at the centre—P—to challenge unsatisfactory options and by extension, genuine and effective person-centredness, is quite limited (a situation also seen in the appeal mechanisms within Deprivation of Liberty Safeguards^99^ and the Mental Health Act).^100^ Claims that arrangements breach Articles 5 or 8 of the ECHR^101^ (or indeed, amount to wrongful detention or false imprisonment)^102^ are available to adults; however, the bar for success in such situations is unsurprisingly high, often resting on demonstrable failings by the relevant public body.^103^ Furthermore, the judiciary has observed that it is not its function to ‘adjudicate on the adequacy of [P’s] care whenever a family member thinks that the quality of local services ought to be better’.^104^ DJ Eldergill observes:

*What can be provided [to those lacking capacity] will still be subject to the same framework of statutory duties and budgetary constraints. To be blunt, their local services will be as stretched and under-funded as they were before; care agency staff will often be temporary, unqualified and lowly-paid ….^105^*

As noted above, the negotiations that take place between the parties in a COP case are likely to exert informal weight on substantive rights. The extent of the impact that *N v ACCG*^106^ had on the informal power of the COP in this regard is not completely clear, but it seems fair to suggest that the knowledge that the COP is limited in what it can order strengthens the negotiating position of a public body. Within the bounds of this caveat, it remains that any option over and above what had been provided initially is more likely than not to become available during that process, especially if that alternative requires fewer resources.^107^ Beyond this informal mechanism, the correct route to challenge substantive failings is an application for judicial review, which, while certainly possible on behalf of persons who lack capacity, can be complex.^108^ Furthermore, judicial review can be costly and, given its limited effectiveness in challenging cuts in other services,^109^ may have a low prospect of success.^110^

Outside of the courts, it may be possible to challenge the care being offered through local authority complaints, given the principles of the Care Act 2014. However, it has been noted^111^ that economically stressed local authorities widely ignore the principles of the Act, and local authority complaints procedures are difficult to mount and ineffective at forcing change.^112^ The House of Lords Adult Social Care Committee has reported that the Care Act is failing—and will continue to fail—to change practice in the absence of adequate funding.^113^ Accordingly, it is difficult to see how this is a viable route in practice. With all of this in mind, the ability of P to challenge genuinely poor and unsuitable options, namely the substantive aspects of best interests, seems mired in difficulty with limited prospects for a good outcome once negotiations have been exhausted.

### B. Resources, best interests and the Courts 2: procedural aspects

While the law could be viewed as largely unable to address failings in the substantive component of best interests—at least where formal judgments are concerned—those who lack capacity have had greater success in having their rights vindicated when procedural aspects are circumvented or ignored, albeit that such failings may not be overtly attributed to resource shortages.^114^ One way that this ‘success’ has manifested is in the interpretation of the scope of the defences contained in the MCA. Section 5 gives immunity from liability once the decision-maker has taken reasonable steps to establish that P lacks capacity and reasonably believes that their act is in P’s best interests. If the decision-maker restrains P, then the defence is also available, so long as the restraint was intended to prevent P suffering harm and was proportionate to the likelihood and severity of that harm.^115^ The scope of these defences was tested in *ZH*^116^ and *Winspear*.^117^

In *ZH*,^118^ it was argued that no liability should attach for the touching and restraint of an autistic man by police and the failure of the officers to consult with his carers at various stages during the interaction.^119^ The Court of Appeal upheld the findings of the judge at first instance, namely that the police could not rely on the MCA to avoid liability for the failure to consult and that the subsequent forceful restraint and detention of a vulnerable person with significant disabilities was disproportionate, thereby breaching his Article 3, 5, and 8 ECHR rights. Furthermore, the Court upheld the finding that ‘[t]he officers’ responses were “over-hasty and ill-informed”^120^ and thus their conduct was not justified given the level of imminent danger and emergency. This seems to suggest that section 5 defence, while robust, is certainly not immune from scrutiny, particularly with respect to the level of emergency, the urgency of the action of the decision-maker, and the degree of infringement on rights defined in the ECHR. As a result, it could be inferred that where a hasty or poor decision-making process or a failure to consult relevant parties as mandated by the MCA is attributed to resource shortages, similar scrutiny would apply. Similarly, the section 5(2) defence was ruled unavailable in *Winspear*,^121^ which applied the principle that a person with capacity must be involved in the process leading to the completion of a DNACPR^122^ notice unless there is a compelling reason not to,^123^ to a man who lacked capacity and accordingly, to his mother as a person engaged in caring for him and interested in his welfare.^124^ The Court found that failing to consult with Mr Winspear’s mother before completing a DNACPR represented a failure to comply with section 4(7) of the MCA and that the defence contained in section 5(2) was unavailable in the circumstances.^125^

While no public body made the claim that lack of resources led to the actions or decisions for which they sought to rely on the section 5 defence, it is a struggle to see how such an argument would succeed given the view of the Court that ‘[t]he protection of the human rights of … the vulnerable members of our society … is too important and fundamental to be sacrificed on the altar of resources’.^126^ Similar approaches can be seen when public bodies have failed to follow proper procedures for arrangements that may deprive individuals of their liberty, though again lack of resources may not be offered as a reason or direct justification for the failures.^127^

Although potentially unsatisfactory from P’s perspective and that of their family, the difference between the way in which substantive and procedural aspects of best interests are handled by the courts may be understandable for a variety of reasons, for example, English law’s proceduralist leanings and the drafting of the MCA itself. Another such explanation is the deeper conflict between the different legal logics and aims of the ECHR and the UNCRPD that we signalled at the beginning of this article. While the Supreme Court views the ECHR and the UNCRPD as consistent in viewing rights as having a ‘universal character’,^128^ this view ignores fundamental differences between the *types* of equality envisioned by the two instruments. The ECHR originated as an ‘anti-statist impulse’^129^ of drafters who sought to curb the perceived state overreach in the creation of post-war welfare states, consciously omitting welfare rights during its drafting.^130^ As challenges to austerity showed, the treaty largely continues to be interpreted in a way that is unreceptive to substantive rights.^131^ In contrast, the UNCRPD is framed around a model^132^ of positive rights,^133^ and many of those rights are unrealizable without redressing structural inequalities.^134^ As such, the UNCRPD and the ECHR are framed around competing legal logics—or at the very least have quite different aims and origins—and their respective legal statuses maintain a fundamental limitation on person-centredness where resources are concerned. *N v ACCG^135^* appears consistent with the more fundamental logic of the court’s interpretation of Article 1 of the ECHR, which guided the court’s approach to the very different liberties framed in *Cheshire West*.^136^  *Cheshire West* was the landmark case that decided the definition of deprivation of liberty. Therein, the court viewed human rights as marked by their ‘universal character’,^137^ pointing to the fact that Article 1 of the ECHR holds that contracting states must ‘secure to everyone’ the convention rights. *Cheshire West* thus holds that P is procedurally equal to everyone else. By that same measure of equality, *N v ACCG*^138^ suggests that P’s equality means they cannot command more resources than anyone else to make their equality substantive. Arguably, this marks out the interpretation of Articles 5 and 8 as fundamentally negative liberties. We suggest this interpretation creates potential for friction between ECHR rights and UNCRPD rights, because it steers best interests decisions away from the idea of equality espoused by the latter.

While the differentiation of procedural and substantive aspects of best interests may be understandable, the impact of this distinction upon people who lack capacity and those who care for and about them is undeniable. It is questionable if the people for whom best interests decisions are made—or their families—would draw as sharp a distinction between the substantive and procedural aspects of the best interests process when both have the potential to impact so significantly on the person. As was stated in the introduction, our starting point is that the best interests process must be thorough and person-centred and allow the most suitable option to be decided upon for P. Our empirical data show that neither the process nor the outcome is satisfactory in some situations, because the resources are not present to make this so.

#### C. Resource Limitations and the Challenge to Person-centredness

Supported by our empirical data, it is our position that at various stages of the best interests process, the person is not central in the way that they should be if a holistic understanding of the term is used.^139^ While it is beyond the scope of this contribution to engage with definitional debates surrounding the meaning of person-centredness more fully, for our purposes, person-centredness can be understood in terms of P’s participation in a decision-making process that reflects P’s individuality, values, and preferences. This entails approaches that are underpinned by recognising P’s inherent value as a person and the provision of options that maximize P’s subjective health, welfare and wellbeing.^140^ Our data suggests, however, that important conversations with P and their trusted people, which are needed for a proper decision-making process, can be cut short. The options provided for P’s care and treatment can be unsatisfactory and seemingly unrelated to what should be available if P’s needs and wishes were a primary consideration. Commentators have also remarked upon the (lack of) participation of P in the COP, a venue where P should certainly be central.^141^

While much of the focus of resource intensity in adult matters has fallen upon Deprivation of Liberty—particularly after the *Cheshire West*^142^ (and perhaps to a lesser extent *Re D*)^143^ rulings—our data show that resource limitations mark every stage of the best interests decision-making process. Participants’ narratives suggest that resources affect everything from admission, treatment decisions and discharge to the way in which best interests decisions are made by the Court. While the increased scrutiny of potential litigation may push decision-makers to be more person-centred, we question the ability of the current model of available choices to deliver person-centred care where those choices are significantly curtailed by resource constraints, sometimes amounting to the ‘least worst option’ rather than the best.

In the context of best interests determinations, the economically bounded nature of decisions is in tension with the person-centred role of the courts to give the person a voice in order that they make ‘the choice which is right for [them] as an individual human being’.^144^ The focus on available choices suggests that best interests decisions must ultimately be constrained by political and economic policy, rather than driven by inherent human value. Thus, it highlights a fundamental clash between the economic and political on the one hand and empowerment and enablement on the other. While we have pointed out some of the distinctions between the ECHR and UNCRPD, we should emphasize that the finitude of resources is not ignored in the latter. Article 4(2) acknowledges that empowerment is bounded by ‘available resources’ and resources can justifiably be withheld on the grounds of public interest because states may use a defence of economic necessity against criticism of funding decisions.^145^ While any failure of best interests decisions to be person-centred may therefore be viewed as arising from immutable economic facts (a view espoused by at least one of our participants), the factual, rather than ideological basis of this state of affairs is open to question. UK austerity policies have been widely recognized as ideological,^146^ since they disproportionately cut support for certain sections of society,^147^ including persons with disability.^148^ As O’Sullivan and McNamara observe, an Article 4(2) defence can only be used when states are forced to limit empowering measures by matters beyond their control, rather than doing so due to ideological motivation.^149^

While it might be objected that such discriminatory policies are the prerogative of a democratically elected executive, such a defence intuitively feels precarious in the face of a challenge based on fundamental rights. Ronald Dworkin’s formula is apposite:

*Individuals have rights when, for some reason, a collective goal is not a sufficient justification for denying them what they wish, as individuals, to have or to do, or not a sufficient justification for imposing some loss or injury upon them.’*^150^

On this view, it is not a necessary feature of human rights to uphold democracy, whereas it is a necessary feature that they uphold the inherent value of individuals—they are human rights, not societal rights. In short, while discriminatory austerity policy was passed democratically by a parliamentary majority, it is precisely the function of rights to protect the liberties of minorities from the depredations of majority interests, even when these interests are democratically expressed. As national and international law are presumed to be harmonious, the precedence of disability rights may offer hope of an influence on the development of the common law.^151^ As yet, that influence seems clearer in terms of procedural aspects of the law than on the substantive.

The resources that are allocated to the best interests process are—and are likely to remain—nominal. We argue that this, at least in part, may be due to a failure to recognize that the best interests process itself is resource-intensive. As suggested by our data, an individualized and person-centred best interests process takes professional time.^152^ This is distinct from the resources needed to provide person-centred care and options. So long as this situation remains, the ‘least worst option approach’ will predominate, falling far short of a person-centred approach. Indeed, assuming this is not already the case, cash-strapped authorities and commissioners faced with financial ruin may simply choose not to offer options, relying on the improbability of legal action being taken.^153^

## VI. CONCLUSION

In her study of 1930s austerity, Clara Mattei concludes that:

*It is a trope of twentieth- and twenty- first- century life that governments faced with financial shortfalls look first to the services they provide their citizens when making cuts. Instances like these are innumerable and span every country in the world. When this happens, they produce highly predictable, uniformly devastating effects on societies.^154^*

Mattei suggests that, historically, this ‘austerity effect’ amounted to a profound erosion of human rights. This was a result of concomitant moralization of poverty and wealth by ideology disguised as immutable economic laws. The parallels with the current situation are striking. Austerity has been accompanied by an increasing hardening of the executive against previously influential liberal themes of human rights and social justice. We suggest that our exploration of best interests through the eyes of individuals, families, and professionals gives some insight into the impact of austerity on best interests decision-making and the inability of individuals to successfully challenge that impact.

Best interests decisions involve putting the person at the centre of the process and giving weight to their wishes and feelings.^155^ However, our data exposes that the person-centred approach to best interests is a resource-intensive process, whether those resources are required to complete an individualized inquiry or to provide suitable options for care or support, yet the law faces a clash of logics between the ECHR and the nascent influence of the UNCRPD on the interpretation of the MCA. The latitude given to decision-makers means that failure to resource to the degree that is necessary for best interests processes to be person-centred will result in something else. Our findings suggest that, outside the rarified world of legal proceduralism, the process at best will be hasty and less considered, irrespective of how honourable professional intentions are, and at worst it can result in apparent collapses in the quality of care, with questionable routes available to resolve failings. In these cases, the approach to best interests is pushed towards a less or non-person-centred process, exposing interpretation not just as a normative, but an economically bounded choice.

Freedom is not free. Discussion of the MCA is conspicuous in being accompanied by a narrative replete with statements of the value of person-centredness, but impoverished of serious action to address the financial costs that these values entail. The extension of liberal freedoms to hitherto disenfranchised segments of the population is a laudable goal of liberal reform, yet there is an odd failure to reckon with the financial and in-kind costs of empowerment by policymakers. While we do not suggest that an unlimited welfare budget be made available, we do argue that the failure to recognize the cost associated with the best interests process itself damages patients, families, and professionals—where it compounds the already obvious lack of resources ‘designated’ for treatment and care. Moreover, this myopic position also does damage to the core aspirations of the MCA by impeding a genuinely person-centred approach to best interests. So long as law embraces ‘a basic modesty in how human rights governance intrude[s] into economic affairs’,^156^ person-centred decision-making will remain just another provisional good that is subject to the tides of ideology, and increasingly seen as optional.

## Data Availability

Due to the sensitivity of the data involved, these data are published as a controlled dataset at the University of Bristol Research Data Repository data.bris. Requests for access will be considered by the University of Bristol Research Data Service, who will assess the motives of potential data re-users before deciding to grant access to the data.

## Footnotes

1 It has appeared in popular culture, eg in public commemoration (as an inscription on the Washington D.C. Korean War Veterans Memorial), as a song lyric ( eg by John Anderson), as a political slogan ( eg by Ronald Reagan) and within satire (Trey Parker’s film ‘Team America’).

2 Select Committee on the Mental Capacity Act 2005, *Mental Capacity Act 2005: Post-Legislative Scrutiny* (HL 2013–14, 139) 108.

3 Mental Capacity Act 2005, s 1(6). See *Re B (Capacity: Social Media: Care and Contact)* [2019] EWCOP 3, para 37 wherein it was noted that a finding that somebody lacks capacity—in this case in respect of internet access—can impose ‘a significant restriction upon his or her freedom’.

4 John Coggon and Camilia Kong, ‘From Best Interests to Better Interests? Values, Unwisdom, and Objectivity in Mental Capacity Law’ (2021) 80 Cambridge Law Journal 245.

5
*Re F (An Adult: Sterilisation)* [1990] 2 AC 1.

6 Margaret Brazier and José Miola, ‘Bye-Bye Bolam: A Medical Litigation Revolution?’ (2000) 8 Medical Law Review 85; *Bolam v Friern Hospital Management Committee* [1957] 1 WLR 582.

7 Law Commission, *Mentally Incapacitated Adults and Decision-Making: A New Jurisdiction* Consultation (Law Com No 128, 1993), para 2.14. Law Commission, *Mental Incapacity* (Law Com No 231, 1995), para 3.25. It is worth noting that the Law Commission did not find the two tests to be mutually exclusive when it made its proposals. The Law Commission’s view of best interests as a choice between available options will be discussed in Part V.

8 Law Commission (1995) ibid, para 3.27.

9
*Bolam* (n 6).

10 The argument has been made that the term ‘best interests’ in capacity law is problematic for this very reason because its intended meaning within the MCA is markedly different to how it was historically used and understood within healthcare and furthermore, in law. See Mary Donnelly, ‘Best interests in the Mental Capacity Act: Time to Say Goodbye?’ (2016) 24 Medical Law Review 318, 331.

11 Mental Capacity Act 2005 s 4(4).

12 ibid s 4(6).

13 ibid s 4(7).

14 ‘Welfare’ encompasses a broad range of areas of decision-making such as residence, care, contact, access to social media, etc.

15 ‘(Un)satisfactory’ is to be understood subjectively.

16 William Hunter, ‘Nurses must Work Harder to “justify” Pay Rise, Says Health Secretary’ (*Nursing in Practice*, 10 January 2023) <https://www.nursinginpractice.com/latest-news/nurses-must-work-harder-to-justify-pay-rise-says-health-secretary/> accessed 18 June 2025; Chris Smyth, ‘Salary Rises must be Justified by Better Productivity, says PM’ (*The Times*, 12 December 2024) <https://www.thetimes.com/uk/politics/article/starmer-tells-public-sector-be-more-productive-if-you-want-better-pay-w3wvrh2j0> accessed 16 May 2025; Matthew Lesh, ‘It’s Boom Time for the Lazy Public Sector—and We’ll all Pay the Price Britain must Ditch its High-cost, Low-outcome Model Once and for All’ (*The Daily Telegraph*, 12 June 2024) <https://www.telegraph.co.uk/business/2024/06/12/boom-time-for-lazy-public-sector-well-all-pay-price/> accessed 16 May 2025.

17 Saoirse Mallorie, ‘Staff Shortages: What’s Behind the Headlines?’ (*Kings Fund Blog*, 6 February 2024) <https://www.kingsfund.org.uk/insight-and-analysis/blogs/staff-shortages-behind-headlines> Accessed 16 May 2025.

18 UNISON, ‘Only Enough is Enough “They never listen” The UNISON Campaign for Safe Hospital Staffing in the NHS 2024 report’ (*UNISON*, 2024) <https://www.unison.org.uk/content/uploads/2025/04/701_Only-enough-is-enough-2025-report.pdf> accessed 16 May 2025.

19 The Health Foundation, ‘Public Perceptions of Health and Social Care Polling (Wave 4: May 2023)’ (*The Health Foundation*, September 2023) <https://www.health.org.uk/sites/default/files/2025-04/Public%20perceptions%20of%20health%20and%20social%20care%20May%202023%20%28Wave%204%29.pdf> accessed 16 May 2025.

20 Select Committee on the Mental Capacity Act 2005 (n 2) 108. Various commentators refer to the empowering ethos or aim of the MCA, eg see Emma Cave, ‘Determining Capacity to Make Medical Treatment Decisions: Problems Implementing the Mental Capacity Act 2005’ (2015) 36 Statute Law Review 86; Camilia Kong and others, ‘Judging Values and Participation in Mental Capacity Law’ (2019) 8 Laws 3.

21 Various commentators have discussed aspects of the MCA which may have rhetorical significance if adhered to improperly or in a tokenistic manner. eg see Mary Donnelly, ‘Best Interests, Patient Participation and The Mental Capacity Act 2005’ (2009) 17 Medical Law Review 1. Donnelly discusses issues which may prevent the ‘participative framework in the MCA’ from having ‘more than rhetorical significance’. See also the empirical work of Jamie Lindsay regarding the ‘rhetorical value’ of the participation of P in the Court of Protection: Jamie Lindsay, *Reimagining the Court of Protection* (CUP 2022).

22 Committee on the Rights of Persons with Disabilities, *Report on follow-up to the inquiry concerning the United Kingdom of Great Britain and Northern Ireland* (CRPD/C/GBR/FUIR/1, 22 March 2024); Committee on the Rights of Persons with Disabilities, *Concluding observations on the initial report of the United Kingdom of Great Britain and Northern Ireland* (CRPD/C/GBR/CO/1 3 October 2017); Björn Bremer and Sean McDaniel, ‘The Ideational Foundations of Social Democratic Austerity in the Context of the Great Recession’ (2020) 18 Socio-Economic Review 439; Lydia Morris, ‘The Moral Economy of Austerity: Analysing UK Welfare Reform’ (2016) 67 British Journal of Sociology 97. Later the various legal mechanisms and the interplay—and arguably, conflict—between them will be discussed.

23 Luke Clements, *Clustered Injustice and the Level Green* (Legal Action Group, 2020). eg we acknowledge that those who lack capacity are in a comparatively better position than those who have capacity and additional needs because those under the MCA have two ‘bites of the cherry’ rather than solely relying on judicial review. We maintain that both groups are poorly served by the current system but, given the focus of our project, our argument targets on those without capacity.

24 Explanatory Notes to the Mental Capacity Act 2005, 4.

25 John Coggon, ‘Is Mental Capacity Law Law?’ (2025) 45 Oxford Journal of Legal Studies 1019. Ian Kennedy memorably termed best interests ‘empty rhetoric’, while Hilary Rodham considered best interests in relation to children to be an ‘“empty vessel” into which adult perceptions and prejudices are poured’ in the making of a decision. Ian Kennedy, ‘Patients, Doctors and Human Rights’ in R Blackburn and J Taylor (eds), *Human Rights for the 1990s* (Mansell 1991) 90; Hillary Rodham ‘Children Under the Law’ (1973) 43 Harvard Educational Review 487.

26 Giles Birchley, ‘The Theorisation of “best interests” in Bioethical Accounts of Decision-making’ (2021) 22 BMC Medical Ethics 68.

27
*Re F* (n 5). That medical opinion is the starting point may explain, in part, why a holistic approach to best interests has been viewed by legislators as being insufficiently integrated into the daily practice of some professionals. See Select Committee on the Mental Capacity Act 2005 (n 2) 6.

28
*Bolam* (n 6).

29 Brazier and Miola (n 6) on *Re F*, 92-9.

30 MCA 2005, s 4(6).

31 MCA 2005, s 1(6): ‘Before the act is done, or the decision is made, regard must be had to whether the purpose for which it is needed can be as effectively achieved in a way that is less restrictive of the person’s rights and freedom of action.’

32
*Heart of England NHS Foundation Trust v JB* [2014] EWCOP 342, para 6. In respect of deprivation of liberty, see Hayden J in *CB v Medway Council & Anor (Appeal)* [2019] EWCOP 5: ‘[W]hat is involved here is nothing less than CB’s liberty. Curtailing, restricting or depriving any adult of *such a fundamental freedom* will always require cogent evidence and proper enquiry. I cannot envisage any circumstances where it would be right to determine such issues on the basis of speculation and general experience in other cases. (our emphasis)’ [para 33]. See also *London Borough of Tower Hamlets v PB* [2020] EWCOP 34, para 50: ‘If P has capacity then manifestly the Act does not apply. If P lacks capacity, facilitating compliance with a regime to which he is opposed will always involve the lightest possible touch, the minimal level of restraint or restriction and for the shortest period of time’.

33
*Wye Valley NHS Trust v B* [2015] EWCOP 60.

34 ibid, para 12.

35 The international human rights treaty which seeks to ‘promote, protect and ensure the full and equal enjoyment of all human rights and fundamental freedoms by all disabled persons’.

36 United Nations Convention on the Rights of Persons with Disabilities, art 12(4).

37 CRPD Committee, General Comment No 1: Equal recognition before the law (art 12), 11 April 2014. It is worth stating that there were mixed views amongst WS2 participants regarding how desirable and useful a change in standard from best interests to rights, will and preference would be.

38 It is beyond the scope of this article to interrogate whether best interests is the appropriate standard for making decisions on behalf of those who lack capacity Moreover, there is ample academic literature on this debate. Examples include Piers Gooding, ‘Navigating the “flashing amber lights” of the right to legal capacity in the United Nations Convention on the Rights of Persons with Disabilities: Responding to major concerns’ (2015) 15 Human Rights Law Review 45; Clíona de Bhailís and Eilionóir Flynn, ‘Recognising Legal Capacity: Commentary and Analysis of Article 12 CRPD’ (2017) 13 International Journal of Law in Context 6; Kevin De Sabbata, ‘Dementia, Treatment Decisions, and the UN Convention on the Rights of Persons with Disabilities. A New Framework for Old Problems’ (2020) 11 Frontiers in Psychiatry 571722.

39 W Martin, S Michalowski, T Jütten, Burch M Essex Autonomy Project (2014) ‘Achieving CRPD Compliance is the Mental Capacity Act of England and Wales Compatible with the UN Convention on the Rights of Persons with Disabilities? If Not, What Next?’ <https://autonomy.essex.ac.uk/wp-content/uploads/2021/01/EAP-Position-Paper-FINAL.pdf>.

40 Peter Bartlett, ‘The United Nations Convention on the Rights of Persons with Disabilities and Mental Health Law’ (2012) 75 Modern Law Review 752, 752–3.

41
*Nottingham University Hospitals v Gregory and Ors* [2023] EWCA Civ 1324.

42 Anna Lawson and Lucy Series, ‘United Kingdom’ in Lisa Waddington and Anna Lawson (eds) *The UN Convention on the Rights of Persons with Disabilities in Practice: A Comparative Analysis of the Role of Courts* (OUP 2018) 417–565; The potential for the UNCRPD (and its monitoring Committee) to be influential on domestic cases concerning medical treatment can be seen in cases such as *Battersbee* (albeit in relation to a child). See *Dance and Battersbee v Barts Health NHS Trust* [2022] EWCA Civ 1055, para 26 (iv). While the ground for appeal based on the CRPD was rejected by the Court of Appeal, it did acknowledge that it was ‘clear from paragraphs 39 and 45 of *Aintree* and elsewhere that the approach in domestic law does afford due respect to wishes and feelings in a manner that would be compatible with the principles of CRPD, Arts 10 and 12’. For an analysis of role played by the UNCRPD Committee in *Battersbee*, see Barry Lyons and Mary Donnelly, ‘A Different Kind of Death? *Barts NHS Trust v Dance and Battersbee*’ (2023) 23 Medical Law International 159. There are also cases in which the relevance of UNCRPD rights have been successfully argued. See *Re NP* [2020] EWCOP 44, eg wherein DJ Taylor observes that ‘[t]he Court must also consider the right to live independently and be included in the community’ in line with art 19 of the CRPD and that where there are two options for residence, then ‘the court should prefer the option that avoids a violation of P’s rights under the CRPD, albeit that the CRPD has not been incorporated into English and Welsh law’ (paras 27–9).

43
*LBX v K & Ors* [2013] EWHC 3230 (Fam); *Commissioner of Police for the Metropolis v ZH* [2013] EWCA Civ 69; *Winspear v City Hospitals Sunderland NHS Foundation Trust* [2015] EWHC 3250 (QB).

44 Oliver Lewis and Ann Campbell, ‘Violence and Abuse against People with Disabilities: A Comparison of the Approaches of the European Court of Human Rights and the United Nations Committee on the Rights of Persons with Disabilities’ (2017) 53 International Journal of Law and Psychiatry 45, 56.

45 eg see Mary Donnelly (n 10); John Coggon, ‘Mental Capacity Law, Autonomy, and Best Interests: An Argument for Conceptual and Practical Clarity in the Court of Protection’ (2016) 24 Medical Law Review 396; Alex Ruck Keene and Cressida Auckland, ‘More Presumptions Please? Wishes, Feelings and Best Interests Decision-making’ (2015) 5 Elder Law Journal 29; The debate extends to the compatibility with freedom of the concept of ‘mental capacity’ itself. See Eilionoir Flynn and Anna Arstein-Kerslake, ‘Legislating Personhood: Realising the Right to Support in Exercising Legal Capacity’ (2014) 10 International Journal of Law in Context 81.

46
*Winspear* (n 43); *LBX* (n 43).

47
*North Yorkshire CC v MAG & Anor* [2016] EWCOP 5.

48 Simon Bottery and Saoirse Mallorie, ‘Social care 360’ (*The Kings Fund*, March 2024) <https://assets.kingsfund.org.uk/f/256914/x/10453129ad/social_care_360_2024.pdf> accessed 18 July 2025; Equality and Human Rights Commission, ‘Seven Years On: disabled people’s rights to independent living, employment and standard of living in the UK’ (*Equality and Human Rights Commission*, August 2023) Available for download at: <https://www.equalityhumanrights.com/our-work/our-human-rights-work/monitoring-and-promoting-un-treaties/crpd/progress-disability-rights> accessed 18 July 2025; Georgina Sturge, Joseph Robins Yago Zayed and Alexander Bellis, *Debate Pack: The Spending of the Ministry of Justice* (CDP-2019-0217) <https://commonslibrary.parliament.uk/research-briefings/cdp-2019-0217/> accessed 18 July 2025; David Haslam, *Side Effects: How Our Healthcare Lost Its Way and How We Fix it* (Atlantic Books 2022).

49 Rowena Crawford, George Stoye and Ben Zaranko, ‘Long-term Care Spending and Hospital use among the Older Population in England’ (2021) 78 Journal of Health Economics 10247.

50 Bottery and Mallorie (n 48).

51 ibid.

52 Tom Harris, Louis Hodge and David Phillips, ‘English Local Government Funding: Trends and Challenges in 2019 and Beyond’ (*Institute for Fiscal Studies*, 13 November 2019) <https://www.econstor.eu/bitstream/10419/235055/1/R166.pdf>.

53 Anushka Asthana, ‘Councils in the North most at Risk Amid Warnings that 127 could Fail Financially in Next Five Years’ (*ITV* 28 February 2024) <https://www.itv.com/news/2024-02-27/127-councils-could-collapse-with-those-in-the-north-of-england-most-at-risk> accessed 20 January 2025.

54 eg the Open Justice Court of Protection Project describes an unreported COP case where the relevant ICB had ceased funding a care home place for a man with a progressive neurological disorder and the local authority had cited the high cost of the care home as the reason why they were unwilling to fund his care indefinitely and why they may withdraw their funding before any the ICB decision could be appealed or a new assessment completed for Mr B His residence in the care home had been judged to be in his best interests at a hearing 3–4 months previously. See: Daniel Clark, ‘When P’s Best Interests aren’t in a Council’s Financial Interests’ (Open Justice Court of Protection Project, 21 April 2024) <https://openjusticecourtofprotection.org/2024/04/21/when-ps-best-interests-arent-in-a-councils-financial-interests/> accessed 16 October 2025.

55 Michael Marmot, ‘Health Equity in England: The Marmot Review 10 Years’ (2020) 368 BMJ m693.

56 Haslam (n 48).

57 Committee on the Rights of Persons with Disabilities, *Concluding observations* (n 22) paras 24, 58(a); Committee on the Rights of Persons with Disabilities, *Report* (n 22); Melina Aikaterini Malli and others, ‘Austerity and the Lives of People with Learning Disabilities: A Thematic Synthesis of Current Literature’ (2018) 33 Disability & Society 1412. See also the final report of the NIHR Impact of Austerity Project: Rachel Forrester-Jones and others, ‘Becoming Less Eligible? Intellectual Disability Services in the Age of Austerity’ (*National Institute for Health Research School for Social Care*, 2020) <https://www.sscr.nihr.ac.uk/SSCR-research-findings_RF100.pdf>.

58 Tinshui Yeung, ‘Starmer Climbs Down on Disability Benefits Cuts after Rebellion by Labour MPs’ (*BBC News* 26 June 2025) <https://www.bbc.co.uk/news/live/cj937xylzyet> accessed 18th June 2025.

59 Dearbail Jordan, ‘Chancellor Sajid Javid Declares End of Austerity’ (*BBC News* 4 September 2019) <https://www.bbc.co.uk/news/business-49577250> accessed 20 May 2025.

60 Workstream 2 conducted interviews and Workstream 1 conducted interviews, focus groups and ethics discussion groups.

61 WS1 advertised through established networks and social media. Because many best interests decisions about patients involved a relative, all non-health professional participants were given the option of being interviewed on their own or in a dyad of patient plus relative.

62 WS2 interviewees were identified through a comprehensive review of best interests cases, chambers and solicitors’ practices, social media, and professional referrals (ie snowball sampling). Those identified were invited to participate via direct email contact, via CoPPA (a multi-disciplinary organization whose aims are to consolidate good practice and develop good practice in the Court of Protection) and through social media.

63 Virginia Braun and Victoria Clarke, ‘Reflecting on Reflexive Thematic Analysis’ (2019) 11 Qualitative Research in Sport Exercise and Health 589.

64 Here ‘adult’ is used to denote those who fall within the scope of the MCA and therefore includes 16–17 year olds.

65 Theodor Adorno, *Problems of Moral Philosophy* (Rodney Livingstone tr, Polity Press 2000) 176.

66 eg if decisions are to be made under mental health or mental capacity legislation.

67 MCA 2005, ss 35–36.

68 Other participants signalled that re-exploring P’s capacity for different decisions or at different times was a frequent area of departure.

69 MCA 2005, ss 1–4.

70
*A Local Authority v DG & Ors* [2014] EWHC 63 (Fam), para 43. Keehan J stated: ‘[W]hatever the difficulties presented by public funding issues and/or the pressure of work, the court will no longer tolerate the failure of parties to comply timeously with court orders. Those failures simply lead to unacceptable delays in the proceedings which are wholly inimical to the welfare of the children involved.’ This was cited with approval in *Re ND (Court of Protection: Costs and Declarations)* [2020] EWCOP 42, wherein Keehan J awarded costs against a local authority for failing *inter alia* to produce care plans for ND as directed by the court.

71 This should not be conflated with a lack of time in an emergency, where a less detailed best interests process may be justified. eg see the Mental Capacity Act Code of Practice, para 5.39, which pertains to the wishes and feelings of P and accounts for less information being gathered in an emergency situation. See also (fn 70): resources cannot be used to justify procedural breaches relating to orders of the Court.

72
*North Yorkshire CC v MAG & Anor* (n 47).

73
*Wye Valley v B* (n 33). See paras 10–13 wherein Peter Jackson J (as he was) makes a number of statements regarding the rights and freedoms of people with disabilities including: ‘It is, I think, important to ensure that people with a disability are not—by the very fact of their disability—deprived of the range of reasonable outcomes that are available to others’. The importance of giving proper weight to the wishes and feelings, beliefs and values of a person who lacks decision-making capacity, which was articulated in paras 10 and 11 of *Wye Valley* has been restated in relation to a range of best interests decisions including termination of pregnancy *Re AB (Termination of Pregnancy)* [2019] EWCOP 26 and covert medication; *Re A (Covert Medication: Residence)* [2024] EWCOP 19. See also the opinion of Hayden J in *Barnsley Hospital NHS Foundation Trust v MSP* [2020] EWCOP 26 that ‘[t]he objective [of the best interests test and of the s. 4(6) enquiry] is to reassert P’s autonomy and thus restore his right to take his own decisions in the way that he would have done had he not lost capacity’ (para 25). Albeit in relation to capacity, rather than best interests, the courts have also expressed the view that the threshold ought not to be set ‘too high’ so as to avoid ‘an unfair, unnecessary and indeed discriminatory bar against the mentally disabled’ (*Sheffield City Council v E* [2004] EWHC 2808 (Fam), which related to marriage. See also *PH v A Local Authority* [2011] EWHC 1704 (Fam) in relation to residence.

74 Alex Ruck Keene, ‘Book Review: Recognising Human Rights in Different Cultural Context: The United Nations Convention on the Rights of Persons with Disabilities (CRPD), Edited by Emily Julia Kakoullis and Kelley Johnson (London: Palgrave Macmillan, 2020)’ (2020) 26 International Journal of Mental Health and Capacity Law 1, 55–56.

75 eg Department of Health, Prime Minister’s challenge on dementia 2020.

76 States parties can advance financial justifications for rights limitations, although Gerards indicates the Strasbourg courts tends to be seen through the prism of negative rights. In cases originating in the UK, Ewing has pointed to the willingness of the court to avoid making decisions with politically sensitive social policy implications in the face of government hostility to the court. We are grateful to our anonymous peer reviewer for this observation and the one at fn 85. See: Janneke Gerards, ‘The ECtHR’s Response to Fundamental Rights Issues Related to Financial and Economic Difficulties the Problem of Compartmentalisation’ (2017) 33 Netherlands Quarterly of Human Rights 274; KD Ewing, ‘Contesting Austerity: The Role of Trade Unions in the UK’ in Farahat Anuscheh and Xabier Arzoz (eds), *Contesting Austerity: A Socio-Legal Inquiry* (Hart 2021) 192–211.

77 Select Committee on the Mental Capacity Act 2005 (n 2) 2. More generally, the Committee noted that the MCA was not embedded in daily practice, noting ‘a general lack of awareness of the provisions of the Act’ and that ‘[t]he empowering ethos of the Act’ had not been widely implemented at that time.

78 ‘Perplexing’ inasmuch as the House of Lords Committee’s position appears to acknowledge resource pressures in practice— eg in the care sector—in other parts of the report, yet appears to us to maintain a moralising view of resource-led decision-making being the norm. If the intention was always that decisions under the MCA would be bounded by resources, then the comments seem curious to us.

79 With that said, our data does suggest that this lack of formal remit to instruct public bodies as to what to do did not necessarily mean that a public body did not change its initial decision once legal proceedings had been commenced. For example, one of our participants suggested: ‘[T]here’s no getting away from it, you can get public authorities to do things they were not otherwise willing to do through Court of Protection … proceedings and back then through the inherent jurisdiction of the High Court.’ (222)

80 Law Commission (1995) (n 7) para 8.19.

81
*N v ACCG and Ors* [2017] UKSC 22.

82 ibid 35.

83 ibid 37.

84 ibid 37.

85 ibid 44.

86 eg some of the empirical data gathered from lawyers suggests that the legal process could sometimes be used successfully to secure better options and outcomes for individuals (see fn79). eg 222 remarked that ‘radically different care arrangements’ could be secured for their clients through the inherent jurisdiction of the High Court—and latterly the Court of Protection—because ‘dragging a local authority before … a High Court judge just seemed like such a powerful thing’ and that local authorities ‘definitely responded to that’. Other lawyers gave ‘softer’ examples of formal best interests processes facilitating, improving or testing the quality of decision-making, which in turn resulted in better outcomes for clients. We explore this in more detail in an upcoming book chapter: Aoife Finnerty and Giles Birchley, ‘Resources in Best Interests—Are they really the “immovable object”?’ in Judy Laing and Sheelagh McGuinness (eds), *Hidden Law Making in the Court of Protection: Perspectives on the Mental Capacity Act* (Bristol University Press) (forthcoming, 2026).

87 B Clough, ‘New Legal Landscapes: (Re)Constructing the Boundaries of Mental Capacity Law’ (2018) 26 Medical Law Review 246, 268.

88
*N v ACCG* (n 81) at [30].

89 Above (n 80).

90
*R v Gloucestershire County Council & Anor, ex parte Barry* [1997] UKHL 58; [1997] AC 584, 604.

91
*A v Liverpool City Council* [1982] AC 363.

92 Alex Ruck Keene and Chiara Cordone, ‘Distributed Rights Protection: The 2000 Hague Convention on the International Protection of Adults and the Challenge of Securing Fundamental Rights Across Borders’ (2023) 91 IJLP 101936.

93 eg the materiality of a public/private law distinction has been a subject of wide-ranging debate, with critics arguing the distinction arises from a scholarly focus on formal avenues of accountability and control, distracting from more penetrating examination of informal mechanisms at work in law. See Peter Cane, ‘Accountability and the Public Private Distinction’ in Nicholas Bamforth and Peter Leyland (eds), *Public Law in a Multi-Layered Constitution* (Bloomsbury 2003) 299–333.

94
*N v ACCG* (n 81), para 29. See also *R (Burke) v General Medical Council* [2005] EWCA Civ 1003, para 50; *Aintree University Hospitals NHS Foundation Trust v James* [2013] UKSC 67, para 18.

95
*North Yorkshire CC v MAG & Anor* (n 47); *N v ACCG* (n 81).

96 It is worth acknowledging the tension inherent in a system where those who challenge decisions may end up getting better options based on the increased scrutiny than those who do not mount a legal challenge, however, it is beyond the scope of this article to engage further with this.

97
*North Yorkshire CC v MAG & Anor* (n 47); *N v ACCG* (n 81), para 37. See also: *R (on the application of McDonald) (Appellant) v Royal Borough of Kensington and Chelsea (Respondent)* [2011] UKSC 33.

98 Asthana (n 53); Emma Dowling, *The Care Crisis: What Caused It and How Can We End It?* (Verso Books 2020); Chris Ham, ‘The Rise and Decline of the NHS in England 2000-20’ (*The Kings Fund*, 2023) <https://www.kingsfund.org.uk/sites/default/files/2023-04/Rise_and_Decline_of_the_NHS_April_2023.pdf> accessed 18th July 2025; Giles Sheldrick, ‘Expert Calls for Urgent Summit to Tackle “worst crisis in 75 years of the NHS”’ (*Daily Express*, 1 May 2023) <https://www.express.co.uk/news/uk/1764985/nhs-crisis-emergency-summit-karol-sikora> accessed 18 July 2025.

99 Lucy Series, Phil Fennell and Julie Doughty, *The Participation of P in Welfare Cases in the Court of Protection: Report for the Nuffield Foundation* (Cardiff University 2017).

100 Sarah Markham, ‘First-tier Tribunals for Mental Health in England: Are they Fit for Purpose?’ (2020) 7 The Lancet Psychiatry 226–27. Our thanks to one of our anonymous reviewers for this observation.

101
*G v E & Ors* [2010] EWHC 621 (Fam); *London Borough of Hillingdon v Neary & Anor* [2011] EWCOP 1377. It is worth noting that while arts 5 and 8 are frequently cited together in this contribution, there is a difference in their effectiveness in this particular area, with art 5 offering much stronger procedural and—at least theoretically—substantive, protections than art 8.

102 See *LB Haringey v Emile* [2020] MHLO (CC).

103 eg in *Neary* (n 101), the person at the centre of the case was taken from his home and placed in respite care, however the local authority indefinitely extended the detention without consent or a court order. In *Emile*, the local authority claimed for unpaid care home fees between 2013 and 2017 in respect of Ms Emile but had taken no steps to authorize her deprivation of liberty.

104
*PB v RB & Anor* [2016] EWCOP 12, para 14.

105 ibid. He continued: ‘There is little the court can do to resolve these dissatisfactions, *which have more to do with central and local government decisions about what resources can or should be made available to individuals* than with the court deciding what to receive on their behalf’ (emphasis added).

106
*N v ACCG* (n 81).

107 eg because the authority has been too risk averse in assessing the staffing levels needed for P’s care.

108 As capacity is decision-specific P may have litigation capacity but lack capacity in other domains such as finance, medical treatment, etc. Where P has litigation capacity, they can seek legal advice, instructing their own solicitor to pursue a judicial review of the decision. If P lacks capacity, in principle, it will be a best interests decision by their litigation friend as to whether judicial review should be pursued. In practice, the decision to pursue judicial review will depend on the merit of the proposed judicial review, and whether P is eligible for legal aid, or whether an alternative form of funding is available for the claim. We extend our thanks to Mathieu Culverhouse of Irwin Mitchell for giving us an insight into how judicial review would operate in practice in these kinds of cases.

109 Veronika Fikfak, ‘Protecting Human Rights in Austerity Claims in the UK’ (2016) 8 Hague Journal on the Rule of Law 205.

110 For further analysis of legal attempts challenge the impacts of austerity policy, including on those with disability, see Charles O’Sullivan and Donna McNamara, ‘The “Necessity” of Austerity and its Relationship with the UN Convention on the Rights of Persons with Disabilities: A Case Study of Ireland and the United Kingdom’ (2021) 21 Human Rights Law Review 157; Ewing, ‘Contesting Austerity’ (n 76).

111 Equality and Human Rights Commission, ‘Challenging Adult Social Care Decisions in England and Wales’ (2023) <https://www.equalityhumanrights.com/sites/default/files/2023/inquiry-challenging-adult-social-care-decisions-in-england-and-wales-report.pdf> accessed 18 July 2025.

112 Reports include unresponsiveness or adversarial responses from local authority officers, restricted access to advocates, lack of account within the process for ongoing illness and disability of the person making the challenge, lack of positive change when challenges were accepted and, rather unsurprisingly in the light of these complaints, reluctance among clients to initiate challenges. See Equality and Human Rights Commission ‘Experiences of challenging adult social care decisions: In-depth interviews’ (2023) <https://www.equalityhumanrights.com/sites/default/files/2023/research-experiences-of-challenging-adult-social-care-decisions-in-depth-interviews_0.docx> accessed 18 July 2025.

113 Adult Social Care Committee, *A ‘gloriously ordinary life’: spotlight on adult social care* (HL 2022-23, 99).

114 The courts have also taken positive steps to promote good procedural practice, eg keenly promoting elaborations of best interests procedure, see LBX (n 43).

115 MCA 2005, s 6.

116
*ZH v The Commissioner of Police for the Metropolis* [2012] EWHC 604 (QB); *ZH* (n 43).

117
*Winspear* (n 43).

118
*Commissioner of Police for the Metropolis v ZH* (n 116).

119 As provided in s 4(7) of the MCA.

120
*Commissioner of Police for the Metropolis v ZH* (n 116).

121
*Winspear* (n 43).

122 Do Not Attempt CPR.

123 The principle was first applied to a person with capacity in *R (Tracey) v Cambridge University Hospitals NHS Foundation Trust and another (Equality and Human Rights Commission and another intervening)* [2014] EWCA Civ 822.

124 MCA 2005, s 4(7)(b).

125 The judge also found a procedural breach of Mr Winspear’s art 8 rights.

126
*Birmingham City Council v D* [2016] EWCOP 8, para 138. Keehan J rejected a claim from the local authority that the resource implications of a ruling that parents could not consent to deprivation of liberty on behalf of 16 and 17 year olds should lead him to find the contrary. See also *Re NRA and Others* [2015] EWCOP 59, para 25 wherein Charles J stated that it was ‘unlikely that a failure to meet the [deprivation of liberty] safeguards could be successfully defended simply on grounds of lack of resources’. See also *J v Bath and North East Somerset Council & Ors* [2025] EWCA Civ 478; *Re X (Court of Protection Practice)* [2015] EWCA Civ 599.

127 eg see *Neary* (n 101) where the local authority was held to have breached Steven Neary’s rights by failing to refer the situation to the Court of Protection and appoint an IMCA for Steven in a timely fashion and for failing to review the best interests assessment (DOL) per the MCA.

128
*P v Cheshire West & Chester Council & another and P & Q v Surrey County Council* [2014] UKSC 19, para 36.

129 Marco Duranti, *The Conservative Human Rights Revolution: European Identity, Transnational Politics, and the Origins of the European Convention* (Oxford Academic 2017) 399.

130 ibid.

131
*Unite the Union v UK* [2016] ECHR 1150; *RMT v UK* [2014] ECHT 366; *Prison Officers Association v UK* App no. 59253/11.

132 The underlying model of disability adopted by the UNCRPD is strongly contested. The social model of disability—the idea that disability is created by the barriers society puts in the way of the full participation of persons with disabling conditions—was central to some understandings of the convention at the time of its adoption. Yet critique of the social model—in particular that it ignores the phenomenology of living with impairment—has led to the observations of the UNCRPD committee more regularly using the ‘human rights model of disability’. See Michael Ashley Stein, ‘Disability Human Rights’ (2007) 95 California Law Review 75, 88–91. Rosemary Kayess and Philip French, ‘Out of Darkness into Light: Introducing the United Nations Convention on the Rights of Persons with Disabilities’ (2008) 8 Human Rights Law Review 1, 7; Theresia Degener, *Routledge Handbook of Disability Law and Human Rights* (Routledge 2016) Chapter 3, 31–49; Jeffrey Miller, ‘The European Disability Rights Revolution’ (2019) 44 European Law Review 66, 69.

133 Michael Ashley Stein and Penelope JS Stein, ‘Beyond Disability Civil Rights’ (2007) 58 Hastings Law Journal 1203.

134 eg rights to independent and community living (art 19), accessibility (art 9) reasonable accommodation (arts 2 and 5) and inclusive education (art 24) can only be realized through the targeted provision of resources to those with disability.

135
*N v ACCG* (n 81).

136
*Cheshire West* (n 128).

137 ibid, para 36.

138
*N v ACCG* (n 81).

139 As Kong argues, there is a longstanding lack of clarity as to the meaning of person-centredness. See Camilia Kong, ‘The Phenomenology and Ethics of P-Centricity in Mental Capacity Law’ (2023) 42 Law and Philosophy 145.

140 Health, welfare and wellbeing are powerful motivators for professionals, but, taken as objective measures, each have histories of being used in ways that oppress persons with disability. We thus signal they should be considered in a subjective sense—ie as they relate to a particular person—as a bulwark against this tendency.

141 eg see Lindsey (n 21); Series, Fennell and Doughty (n 99).

142
*Cheshire West* (n 128). By broadening the definition of deprivation of liberty, thereby impacting considerably on public authorities, it led to a significant increase in workload for local authorities because of the increased numbers of people whose arrangements would now amount to deprivation of liberty. For concerns about resources, see Eve Piffaretti and Richard Jones, *Mental Capacity Act Manual* (Sweet & Maxwell 2018).

143
*Re D (A Child)* [2019] UKSC 42.

144
*Aintree* (n 94), para 45.

145 O’Sullivan and McNamara (n 110).

146 See n 22.

147 Laura Gardiner, ‘The Shifting Shape of Social Security: Charting the Changing Size and Shape of the British Welfare System’ (*Resolution Foundation*, 2019) <https://www.resolutionfoundation.org/app/uploads/2019/11/The-shifting-shape-of-social-security.pdf> accessed 18 July 2025.

148 Forrester-Jones and others (n 57); Aikaterini Malli and others (n 57).

149 O’Sullivan and McNamara (n 110).

150 Ronald Dworkin, *Taking Rights Seriously* (Harvard University Press 1978) xi.

151 Lawson and Series (n 42).

152 A similar process can be observed with the under resourcing of the Care Act 2014. As noted previously, the HOL committee suggested that it is ignored by cash strapped authorities who do not change things substantively even if successfully challenged. Adult Social Care Committee (n 113).

153 This possibility was noted by a participant: ‘[T]he best interests options can in practice always be constrained by the local authority or the funder taking a public law decision to take them off the table. Actually, in practice, the local authority and CCG can drive the best interests outcome by making decisions that can’t be challenged in the Court of Protection in any event. And it’s great that most of them don’t do that but the best interests consideration is already operating within an artificially constrained framework’ (228). See also Clough (n 87).

154 Clara Mattei, *The Capital Order: How Economists Invented Austerity and Paved the Way to Fascism* (The University of Chicago Press 2023) 2.

155 Used as shorthand for wishes, feelings, beliefs, values and that which is important to them.

156 Samuel Moyn, *Not Enough: Human Rights in an Unequal World* (Harvard University Press 2018) 194.

